# Variation in practice for preoperative antibiotic prophylaxis: a survey from an academic tertiary referral center in the United States

**DOI:** 10.1186/s13037-021-00308-3

**Published:** 2021-10-27

**Authors:** Nikhil Ailaney, Elizabeth Zielinski, Michelle Doll, Gonzalo M. Bearman, Stephen L. Kates, Gregory J. Golladay

**Affiliations:** 1 Department of Orthopaedic Surgery, Virginia Commonwealth University School of Medicine, 1200 Broad Street, 9th Floor, VA 23298 Richmond, USA; 2 Department of Orthopaedic Surgery, Virginia Commonwealth University Health, VA 23298 Richmond, USA; 3 Department of Infectious Disease, Virginia Commonwealth University Health, VA 23298 Richmond, USA

**Keywords:** Antibiotic prophylaxis, Surgical site infection, Orthopaedic surgery, Quality improvement

## Abstract

**Background:**

Antibiotic surgical prophylaxis is a core strategy for prevention of surgical site infections (SSI). Despite best practice guidelines and known efficacy of antibiotic prophylaxis in decreasing SSI risk, there is often wide variation in its use. This study was designed to determine the individual perspectives of perioperative providers at an academic tertiary referral center regarding their knowledge of preoperative antibiotic choice, dosing, and timing.

**Methods:**

A prospective survey was conducted amongst surgical and anesthesia team members involved in preoperative antibiotic decision making. The survey addressed ten key principles relating to preoperative antibiotic use, including antibiotic choice, timing and rate of infusion, and dosing. The survey was distributed among orthopaedic surgeons, residents, and anesthesia providers at their respective monthly service line meetings between August 2017 to June 2019. The data was stored and analyzed in a Microsoft Excel worksheet.

**Results:**

A total of 73 providers completed the survey. Twenty-two (30 %) of the providers agreed and 47 (64 %) disagreed that both vancomycin and cefazolin are equally effective for antibiotic prophylaxis. As for antibiotic choice in patients with penicillin allergies, 37 (51 %) agreed with vancomycin, 21 (29 %) agreed with clindamycin, and 15 (21 %) disagreed with both alternatives. When providers were surveyed regarding the appropriateness of standard versus weight adjusted dosing, 67 (92 %) agreed that vancomycin should be weight adjusted and 63 (86 %) agreed that cefazolin should be weight adjusted.

**Conclusions:**

There is no clear consensus amongst providers for which antibiotic to administer for antibiotic prophylaxis despite existing guidelines. Discrepancy also exists between orthopaedic surgery and anesthesia providers in regards to appropriate antibiotic choice for patients with reported penicillin allergies. Institutions should implement evidence-based protocols for preoperative antibiotic prophylaxis and continue to prospectively monitor compliance in order to identify any inconsistencies that could result in inappropriate antibiotic prophylaxis for patients.

## Background

Surgical site infections (SSI) continue to be one of the most common complications after orthopaedic surgery [1, 2]. Patients who develop SSI are at an increased risk of morbidity and mortality, often have longer hospital length of stays, and also have greater health care associated costs [3, 4]. One of the most important strategies to reduce the risk of SSI is antibiotic prophylaxis, with a goal of decreasing the overall burden of microorganisms at the operative site [5]. Since the most common pathogen associated with SSI in orthopaedic procedures is Methicillin-sensitive Staphylococcus aureus (MSSA), antibiotics with excellent gram-positive coverage, such as first or third generation cephalosporins are often preferred. However, Methicillin-resistant Staphylococcus aureus (MRSA), Coagulase-negative Staphylococci (CoNS), and gram-negative bacilli are also important pathogens to consider. In addition, patient allergies, the side effect profile, and the cost associated with the antibiotic must also be considered.

The efficacy of antibiotic prophylaxis within the field of orthopaedic surgery is well documented. In total knee and total hip arthroplasty, a study reported an 81 % decrease in risk of SSI with the use of antibiotic prophylaxis compared to without [6]. Similarly, in hip fracture surgery, a study reported almost 50 % reduction in the rate of SSI with the use of antibiotic prophylaxis compared to without [2]. However, despite best practice guidelines and the known efficacy of antibiotic prophylaxis in reducing the risk of SSI, there is evidence of wide variation in antibiotic prophylaxis practices [7–9]. A study involving 2,965 hospitals, including 34,133 patients, determined that only 56 % of patients received antibiotic prophylaxis within 60 min of the incision and another 20 % of the patients received antibiotics between one and two hours before incision [7]. In addition, almost 10 % of the patients received their first dose of antibiotics greater than four hours after the time of incision [7]. The authors also analyzed the time which antibiotics were discontinued and determined that antibiotics were discontinued within 24 h in only 41 % of the patients studied [7].

Considering the effectiveness of antibiotic prophylaxis for decreasing the risk of SSI but the potential for great variability in its use despite best practice guidelines, we performed a qualitative study to assess the antibiotic prophylaxis perspectives of the orthopaedic surgery and anesthesiology teams at Virginia Commonwealth University (VCU) Health regarding preoperative antibiotic choice, dosing, and timing.

## Methods

**Fig. 1:**
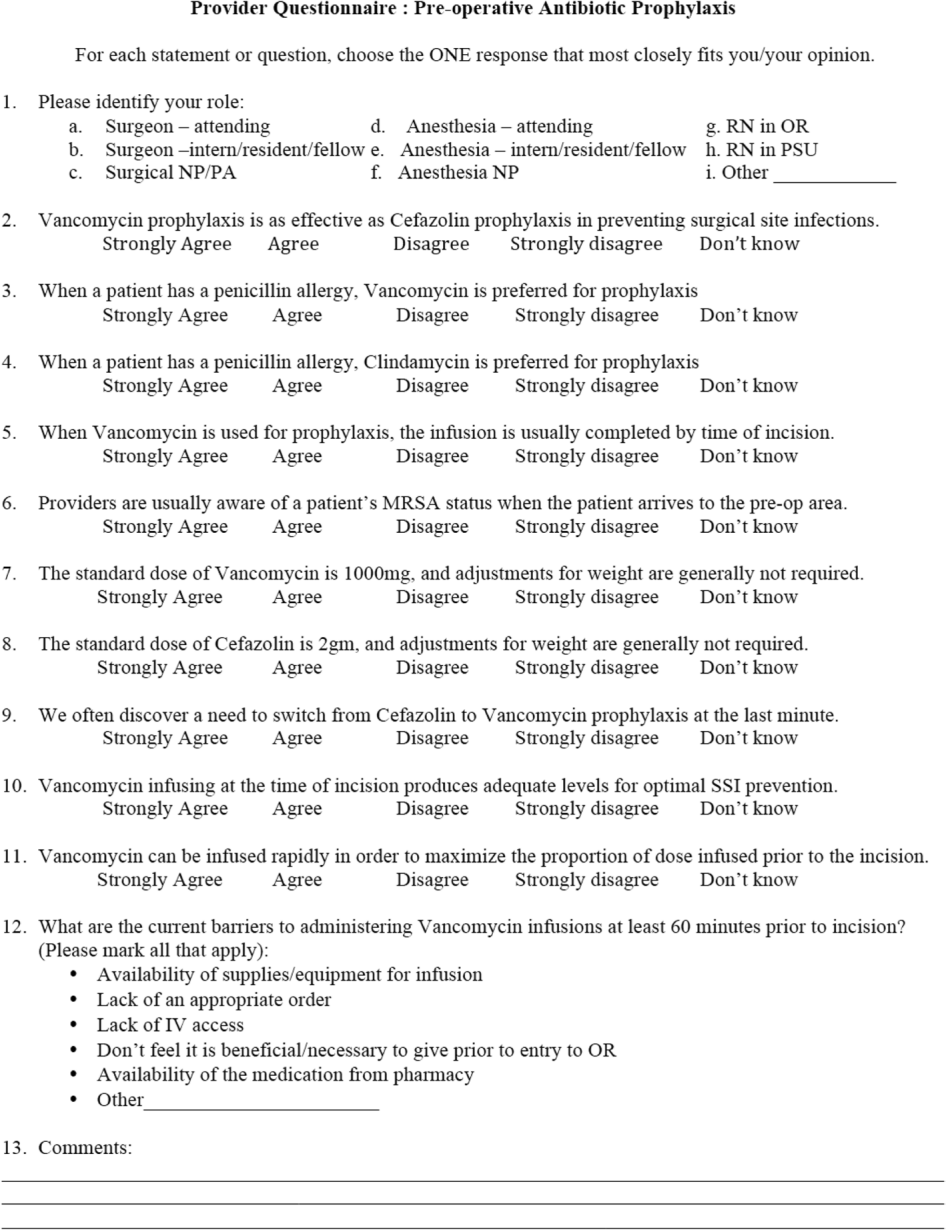
Provider survey regarding preoperative antibiotic prophylaxis

This study was conducted at an 850-bed tertiary care hospital with institutional pre-operative prophylaxis guidelines in place that prefer cefazolin with vancomycin as an alternative for penicillin allergy or an addition for MRSA-colonized patients. An Institution Review Board (IRB) approved survey (Fig. 1) was distributed amongst both orthopaedic surgery (nurse practitioners (NP’s), resident physicians, and attending physicians) and anesthesia (certified registered nurse anesthetists (CRNA’s), resident physicians, and attending physicians) team members involved in preoperative antibiotic decision making from August 2017 to June 2019. The survey was distributed to all providers that met inclusion criteria during one monthly mandatory department meeting and anonymously collected at the end of the meeting. To meet inclusion criteria, providers that completed surveys had to be practicing resident physicians, advanced practice providers (NP’s/CRNA’s), or attending physicians within the orthopaedic surgery or anesthesiology departments at VCU Health. Medical students and ancillary surgical staff (surgical technologists, circulating nurses, and general perioperative nursing staff) were excluded from the study. Orthopaedic surgery residents and NP's were surveyed in August 2017, attending orthopaedic surgeons in October 2018, and anesthesia providers in June 2019. The survey addressed ten key practices relating to preoperative antibiotic use, including antibiotic choice for given clinical scenarios, timing and rate of antibiotic infusion, and antibiotic dosing. In addition, we collected opinions regarding barriers to timely antibiotic administration. After completion of the surveys by providers, the data was stored and analyzed in a Microsoft Excel worksheet.

## Results

### Nurse practitioner and resident orthopaedic surgery providers

**Fig. 2:**
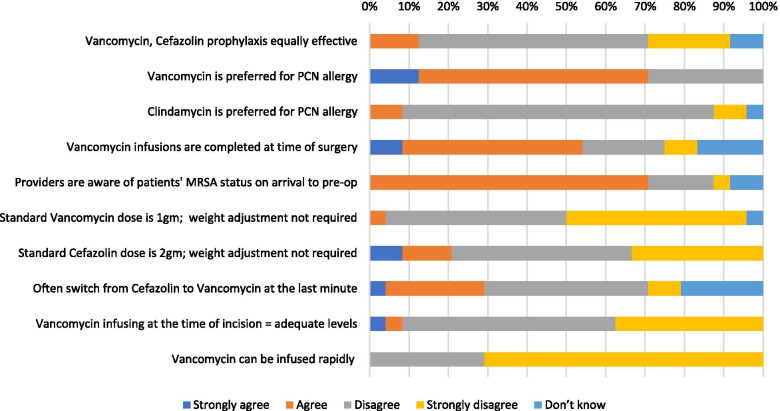
Nurse practitioner and resident orthopaedic surgery provider responses regarding surgical antibiotic prophylaxis

A total of 2 orthopaedic NP’s and 25 orthopaedic surgery residents were approached to complete the survey. Both NP’s (100 %) and 22 (88 %) residents completed the survey. A total of 3 providers (13 %) agreed that vancomycin and cefazolin are equally effective for antibiotic prophylaxis whereas 19 (79 %) disagreed, and 2 (8 %) were unsure (Fig. 2). As for the antibiotic choice for patients with a penicillin allergy, 17 providers (71 %) agreed with vancomycin as the preferred alternative, 2 (8 %) preferred clindamycin, and 5 (21 %) disagreed with both practices. When providers were surveyed regarding the appropriateness of standard versus weight adjusted dosing, 22 (92 %) agreed that vancomycin should be dose adjusted by weight and 19 (79 %) agreed that cefazolin should be weight adjusted. Specific to vancomycin administration, the results indicated barriers to its effectiveness as a suitable method for prophylaxis. 22 providers (92 %) agreed that vancomycin infusion at the time of incision does not allow for adequate concentrations for appropriate antibiotic prophylaxis. In addition, 24 providers (100 %) recognized that vancomycin cannot be infused rapidly in order to maximize the proportion of dose infused prior to the time of incision. Furthermore, only 13 providers (54 %) agreed that vancomycin infusions are completed at the time of surgery. Common barriers to timely administration of vancomycin prior to incision included issues with the availability of the medication from the pharmacy, the availability of equipment required for infusion, incorrect medication ordering, the lack of intravenous (IV) access for the patient, and other issues with the preoperative nursing staff.

### Attending orthopaedic surgery providers

**Fig. 3:**
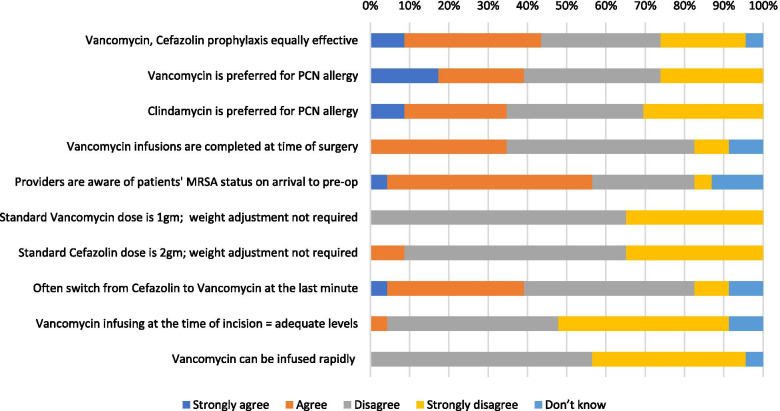
Attending orthopaedic surgery provider responses regarding surgical antibiotic prophylaxis

A total of 28 attending orthopaedic surgeons were approached to complete the survey. Twenty-three (82 %) attending orthopaedic surgeons completed the survey. Ten (44 %) attending orthopaedic surgeons agreed that vancomycin and cefazolin are equally effective for antibiotic prophylaxis whereas 12 (52 %) disagreed, and 1 (4 %) was unsure (Fig. 3). Nine providers (39 %) preferred vancomycin as the antibiotic choice for patients with a penicillin allergy, 8 (35 %) preferred clindamycin, and 6 (26 %) disagreed with both practices. All 23 (100 %) of the attending orthopaedic surgeons surveyed agreed that vancomycin should be dose adjusted by weight and 21 (91 %) agreed that cefazolin should be weight adjusted. The attending orthopaedic surgeon data also indicated barriers to vancomycin’s effectiveness as a suitable method for prophylaxis. Twenty-two (87 %) agreed that vancomycin infusion at the time of incision does not allow for adequate concentrations for appropriate antibiotic prophylaxis. In addition, 22 providers (96 %) recognized that vancomycin cannot be infused rapidly in order to maximize the proportion of dose infused prior to the time of incision. Furthermore, only 8 providers (35 %) agreed that vancomycin infusions are completed at time of surgery. Similar to the NP and resident data, additional reported barriers to timely administration of vancomycin prior to incision included issues with the preoperative nursing or anesthesia staff which delayed administration.

### Anesthesia providers (CRNA’s, Residents, Attendings)

**Fig. 4:**
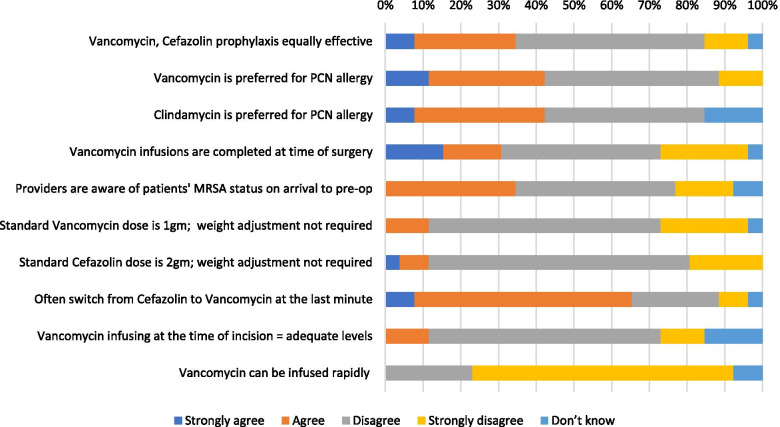
Anesthesia provider responses regarding surgical antibiotic prophylaxis

Twelve CRNA’s, 6 anesthesia residents, and 8 attending anesthesiologists completed the survey. Nine (35 %) anesthesia providers agreed that vancomycin and cefazolin are equally effective for antibiotic prophylaxis whereas 16 (62 %) disagreed, and 1 (4 %) was unsure (Fig. 4). Eleven (42 %) anesthesia providers preferred vancomycin as the antibiotic choice for patients with a penicillin allergy, 11 (42 %) preferred clindamycin, and 4 (15 %) disagreed with both practices. As for the question regarding standard versus weight adjusted dosing, 22 (85 %) anesthesia providers agreed that vancomycin should be dose adjusted by weight and similarly 23 (88 %) agreed that cefazolin should be weight adjusted. Similar to all of the other providers surveyed in this study, the anesthesia provider data also indicated barriers to vancomycin’s effectiveness as a suitable method for prophylaxis. Nineteen (73 %) providers agreed that vancomycin infusion at the time of incision does not allow for adequate concentrations for appropriate antibiotic prophylaxis. In addition, 24 (92 %) providers recognized that vancomycin cannot be infused rapidly in order to maximize the proportion of dose infused prior to the time of incision. Furthermore, only 8 (31 %) providers agreed that vancomycin infusions are completed at time of surgery. Additional reported barriers to timely administration of vancomycin prior to incision included issues with the availability of vancomycin from the pharmacy, the availability of equipment required for infusion, lack of an appropriate order, lack of patient IV access, and issues with the preoperative nursing staff.

### Impact of service and role on survey responses

For most survey questions, a large difference in responses between provider type (residents, attendings, NP’s, or CRNA’s) or service (anesthesia or orthopaedics) was not observed. However, there was significant disagreement regarding preferred agent for penicillin allergies, with residents more frequently indicating agreement with vancomycin (20 out of 28, 71 % agreement) compared to CRNA’s/NP’s (4 out of 14, 29 % agreement), and attendings (13 out of 31, 42 % agreement). There were also differences between anesthesia and orthopaedics regarding awareness of patients’ MRSA status with 30 (64 %) orthopaedic providers in agreement that MRSA status is known and only 9 (35 %) anesthesia providers in agreement. Lastly, anesthesia was more likely to agree that frequent switches from cefazolin to vancomycin occur at the last minute (17 out of 26 (65 %) in agreement versus 16 out of 47 (34 %) amongst orthopaedic providers).

## Discussion

Despite well established guidelines and the known efficacy of antibiotic prophylaxis for reducing the risk of SSI, there continues to be wide variation amongst antibiotic prophylaxis practices. Therefore, we performed this study to assess the perspectives of providers at our institution regarding some of these practices including preoperative antibiotic choice, dosing, and timing. We determined that there is no clear consensus regarding the effectiveness of vancomycin and cefazolin for antibiotic prophylaxis since 30 % of providers agreed and 64 % disagreed that both antibiotics are equally effective. Similarly, there was also no consensus on the antibiotic choice for patients with a penicillin allergy since 51 % of those surveyed agreed with vancomycin, 29 % agreed with clindamycin, and the remaining 21 % disagreed with both alternatives. In contrast, providers did generally agree with necessity of weight based dosing and timely infusion of vancomycin.

Overall, the results from this study indicate that there is no clear consensus amongst the providers at our institution when it comes to which antibiotic to administer for prophylaxis against SSI despite institutional guidelines developed by surgical service leadership. Several institutions from all over the world have determined that antibiotic prophylaxis is often inadequately administered. In a study from China that included 53 hospitals and a total of 14,525 procedures, Ou et al. determined that in only 9.4 % of the procedures was antibiotic prophylaxis appropriate and correct in all steps, which included antibiotic choice, dose, dosing strategy, time of administration and duration of prophylaxis [10]. Similarly, Hawkins et al. performed a study involving 143 pediatric procedures and found that although 99 % of the patients were correctly given or withheld prophylactic antibiotics, complete adherence to antibiotic guidelines was only present in 48 % of cases [11]. In fact, weight-based dosing was present in only 77 % of cases, timing of administration was correct in only 73 % of cases, and only 7 % of cases were appropriately re-dosed [11]. Similarly, in a study from France including 1,312 procedures, Muller et al. determined that non-compliance to the French national recommendations was evident in 44 % of cases they studied [12]. In addition, specific to patients with beta-lactam allergies, Nguyen et al. demonstrated that among the cohort of patients they studied, only 37 % of patients with labeled beta-lactam allergies received appropriate preoperative antibiotic prophylaxis compared to 76 % appropriateness in patients without labeled allergies [13]. Therefore, although well-established antibiotic prophylaxis guidelines exist, great variability and poor compliance are major obstacles to adequate prophylactic antibiotic administration.

The explanations for our results are multifactorial. One reason for the lack of consensus in terms of appropriate antibiotic choice may be due to the fact that best practice guidelines are not widely displayed throughout preoperative and operative areas at our institution. Therefore, lack of awareness could be a potential contributor to our results. Another potential explanation is that since there is no formal education or training for both orthopaedic surgery and anesthesia team members regarding the topic of antibiotic prophylaxis, providers at our institution may not possess the most up-to-date knowledge in regards to this topic. Furthermore, as antibiotic resistance and drug allergies continue to increase in our communities, there is a need to continually educate health care providers on the most current literature available. Thus, an educational gap could be another contributing factor to our results. Lastly, there is also no antibiotic prophylaxis checklist at our institution to help standardize prophylaxis practices, which is often a key component of successful quality improvement initiatives [14].

In contrast to antibiotic choice, there was agreement at our institution that cefazolin and vancomycin dose should be weight adjusted. This consensus is most likely explained by the fact that the electronic medical record (EMR) at our institution prompts physicians to use weight-based dosing when ordering prophylactic antibiotics. We also determined that providers agreed that vancomycin infusion at the time of incision at our institution is often not adequate for antibiotic prophylaxis. This has severe implications because it is well documented that patients with inadequate vancomycin infusion have a significantly higher risk of SSI compared to patients where infusion is complete prior to incision. In a study by Cotogni et al. involving 741 cardiac surgery patients, patients where vancomycin infusion was violated (i.e. surgical skin incision was performed before the end of vancomycin infusion) had greater than five times increased odds of SSI compared to patients where vancomycin infusion was completed prior to incision [15]. Through our survey, we learned that this finding was most likely due to many factors such as problems with antibiotic availability from the pharmacy, missing infusion equipment in the preoperative areas, problems with the preoperative nursing staff, no EMR order, or a lack of patient IV access which delayed the start of antibiotic infusion.

Based on the results from this study, we have determined that there may be many potential areas for improvement at our institution when it comes to antibiotic prophylaxis. However, results of quality improvement programs to improve antibiotic prophylaxis have been mixed. In a study from the University of Texas at Houston, Putnam et al. implemented three cycles of interventions from 2011 to 2014 to improve antibiotic prophylaxis [16]. A few of their interventions included modifying their pre-incision checklist to include all four elements of antibiotic administration (i.e. type, dose, timing, redosing), assigning the anesthesia team the role of antibiotic administration, and distributing and displaying prophylaxis guidelines [16]. After the interventions, the researchers found that although redosing compliance significantly improved, overall adherence and adherence to the correct dose and timing was unchanged. Furthermore, antibiotic type errors significantly increased after the interventions [16]. Similarly, in a study from Australia, Knox and Edye compared preintervention antibiotic prophylaxis practices to compliance after implementation of an interventional program that included displaying prophylaxis guidelines in surgical areas and advertising appropriate prophylaxis practices throughout their institution [17]. After the intervention, the researchers determined that overall adherence was unchanged with adherence at 18 % preintervention and 15 % postintervention [17]. In a study from Canada by So et al., the researchers also compared preintervention antibiotic prophylaxis compliance to postintervention compliance [18]. Their interventions included posting antibiotic protocols in the operating room (OR), having only recommended antibiotics readily available in the OR, educating resident physicians during orientation, including prophylactic antibiotics at time out, and both computerized alerts and emails to physicians when protocols were not followed [18]. In contrast to the studies mentioned above, the researchers found that within the field of orthopaedics, complete compliance to established guidelines drastically increased from 4.5 % preintervention to 54 % postintervention [18]. Furthermore, the greatest improvement was in regards to the duration of antibiotics, where compliance improved from 9.5 % preintervention to 75 % postintervention [18]. Similarly, in a study from Egypt, Saied et al. developed and taught a two-day curriculum designed to educate anesthesiologists and surgeons at five institutions about proper antibiotic prophylaxis practices, specifically focusing on the time and duration of antibiotic administration [19]. The researchers determined that compared to preintervention antibiotic prophylaxis practices, the optimal timing of the first dose significantly improved in three of the five institutions and the optimal duration of prophylaxis improved by 25 % in all five institutions, postintervention [19]. Therefore, based on the studies mentioned above, implementation of a multifactorial quality improvement strategy that includes an educational component may be beneficial to improve antibiotic prophylaxis adherence.

Our study has several important limitations. First, the study is purely qualitative since we gathered provider data with the use of a questionnaire. Second, the purpose of this study was to only assess the antibiotic prophylaxis perspectives at our institution; we did not perform a retrospective analysis to determine the actual adherence to antibiotic prophylaxis guidelines at our institution. Therefore, although we found no clear consensus amongst our providers when it comes to which antibiotic to administer for prophylaxis against SSI, we cannot determine if this finding directly translates to poor adherence to antibiotic guidelines at our institution. Third, our study is subject to selection bias since our sample size of 73 providers is small and we only surveyed providers involved with the care of orthopedic patients. Furthermore, specific to anesthesia providers, although department meetings are mandatory, not all anesthesia providers were present and thus we were unable to approach all anesthesia team members at VCU Health to complete surveys. Lastly, it is important to mention that these results are based on responses from providers only at one institution.

## Conclusions

Our survey indicated that there is no clear consensus amongst providers for which antibiotic to administer for prophylaxis against SSI despite existing internally developed and surgery-type specific guidelines. There is also great discrepancy between orthopaedic surgery and anesthesia providers in regards to appropriate antibiotic choice for patients with reported penicillin allergies. Therefore, based on our results, institutions should implement evidence-based protocols for preoperative antibiotic prophylaxis and continue to prospectively monitor compliance in order to identify any inconsistencies that could result in inappropriate antibiotic prophylaxis for patients.

## Data Availability

The data sets used and analyzed during the current study are available from the corresponding author on reasonable request.

## Abbreviations

SSI: Surgical site infections
MSSA: Methicillin-sensitive Staphylococcus aureus
MRSA: Methicillin-resistant Staphylococcus aureus
CoNS: Coagulase-negative Staphylococci
VCU: Virginia Commonwealth University
IRB: Institution Review Board
NP’s: Nurse practitioners
CRNA’s: Certified registered nurse anesthetists
IV: Intravenous
EMR: Electronic medical record
OR: Operating room
